# Fracture of the Atlas through a Synchondrosis of Anterior Arch

**DOI:** 10.1155/2013/934135

**Published:** 2013-11-14

**Authors:** Gamze Turk, Ismail M. Kabakus, Erhan Akpinar

**Affiliations:** Department of Radiology, Hacettepe University School of Medicine, Sihhiye, 06100 Ankara, Turkey

## Abstract

Cervical fractures are rare in paediatric population. In younger children, cervical fractures usually occur above the level of C4; whereas in older population, fractures or dislocations more commonly involve the lower cervical spine. Greater elasticity of intervertebral ligaments and also the spinal vertebrae explains why cervical fractures in paediatric ages are rare. The injury usually results from a symmetric or asymmetric axial loading. In paediatric cases, most fractures occur through the synchondroses which are the weakest links of the atlas. The prognosis depends on the severity of the spinal cord injury. In this case, we presented an anterior fracture in synchondrosis of atlas after falling on head treated with cervical collar. There was no neurologic deficit for the following 2 years.

## 1. Introduction

Cervical fractures rarely occur in pediatric age group. Since the first description of an atlas fracture in 1822, there have been few reported cases. When a pediatric patient presents with persistent pain and limited movement of the neck and plain radiographs do not demonstrate an abnormality, high resolution computed tomography should be undertaken to assess for minimally displaced fractures through the anterior synchondrosis. Congenital anomalies and ossifications centers can make image interpretation very difficult. Maximum intensity projection (MIP) and volume rendered reconstructions can greatly improve diagnostic confidence. We describe a case of a fracture through the anterior synchondrosis and illustrate the contribution of reconstructed CT images to interpretation.

## 2. Case Report

A 4-year-old girl presented to the paediatric emergency room with neck pain. She had fallen from the bunk bed onto the top of her head 3 days ago. The family described no loss of consciousness or change in mental status. There was no nausea or vomiting. The neurological examination showed no significant neurological deficit even though the patient was slightly agitated. There was a minimal decrement in the range of neck motion and cervical stiffness. The rest of the physical exam was unremarkable.

**Figure 1:**
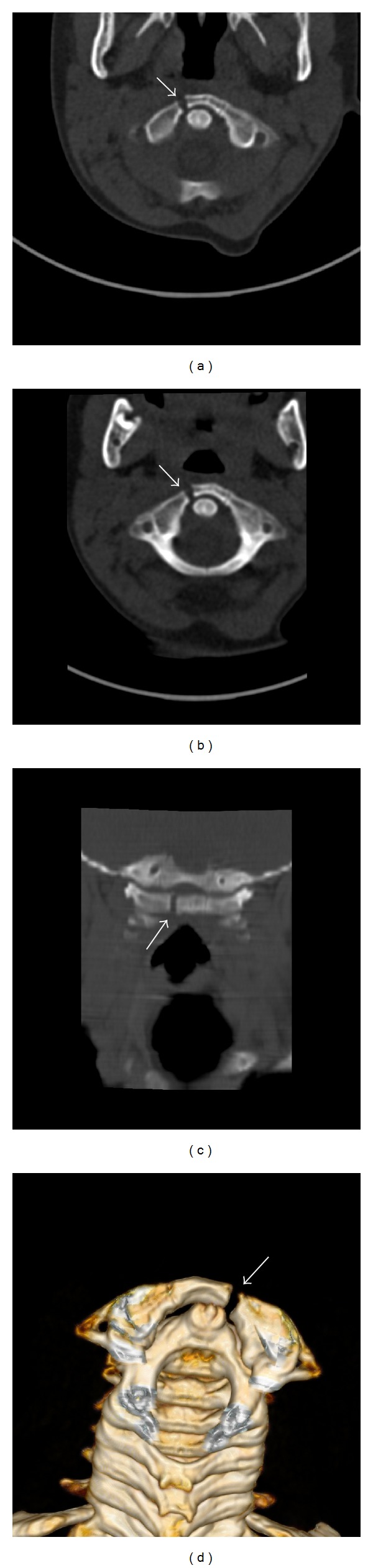
Axial plan, CT scan of cervical vertebrae with 2 mm slice thickness (a), axial oblique (b), coronal oblique (c), MIP reconstruction, and posterior view of 3D volume rendering (d) images show fracture of the atlas through a synchondrosis of the anterior arch (arrows). The space at right synchondrosis line is measured 3.5 mm.

Conventional standard radiographs of the cervical spine did not show evidence of a fracture. As the patient had persistent stiffness, a CT scan of the cervical spine was undertaken. Standard axial images showed slight diastasis (3.5 mm) of the right synchondrosis of anterior arch of the atlas. The relative anatomical relationships are more evident on the coronal oblique MIP and 3D volume rendered reconstructions (Figure 1). There were no signs of rotation or soft tissue hematoma.

The patient had the cervical spine immobilized via a collar and was ordered thiocolchicoside 2 × 2 mg for the symptoms. During her followup, she did not have any neurological deficits.

## 3. Discussion

Injury to the cervical spine occurs infrequently in pediatric population and represents only 1.9% to 9.5% of all cervical injuries. Atlas fractures are also rare among pediatric cervical injuries [1, 2]. Five groups of atlas fractures have been described: isolated fractures of the anterior arch of the atlas, isolated fractures of the posterior arch, combined fractures of the anterior and posterior arches (Jefferson fractures), isolated fractures of the lateral mass, and fractures of the transverse process [3].

Sir Astley Cooper reported the first case at autopsy 1822 in a 3-year-old boy with fractures of both arches of the atlas, and Jefferson described 65 cases in his widely cited article about fractures of atlas [4]. The presence of congenital anomalies as well as the variability of the maturation of the synchondrosis causes pitfalls in the imaging and the diagnosis at paediatric trauma patients. The clinical picture usually consists of neck pain, cervical muscle spasm, head tilt, and decreased range of motion following a fall onto the top of the head [2, 5]. The symptoms are usually short-term and do not require a specific treatment, leading difficulty in diagnosis of cervical fractures even retrospectively.

Cervical fractures are rare in paediatric population. Before eight years of age, cervical fractures usually occur above the level of C4; whereas in older children the fractures or dislocations more commonly involve the lower cervical spine [6]. Greater elasticity of intervertebral ligaments and also the spinal vertebrae explains why cervical fractures in paediatric ages are rare. The injury usually results from a symmetric or asymmetric axial loading. In paediatric cases, most fractures occur through the synchondroses which are the weakest links of the atlas [5].

The prognosis depends on the severity of the spinal cord injury. When the injury does occur, it is associated with a higher mortality rate and is usually in the upper cervical vertebra due to the horizontal orientation of the upper cervical facets in paediatric age group [7]. Neurological deficit is rare in published cases. The cord is probably protected due to the centrifugal displacement of the fracture fragments [2]. Fractures of atlas may be associated with transverse ligament rupture or avulsion, which cause gross atlantoaxial instability. Ruptures of the ligament are best visualized with MRI, whereas atlantoaxial instability is typically evaluated with dynamical cervical spine radiographs [8].

In order to successfully diagnose a pediatric cervical fracture, a high index of suspicion is required in the context of the appropriate clinical history and detailed clinical examination. The patients usually come to the emergency room with neck pain, cervical muscle spasm, and decreased range of motion following a fall onto the head. Since the clinical picture may not be clear, these fractures can be easily overlooked. Making the diagnosis can be very challenging radiologists due to obscure clinical findings and pittfalls. The possible variants such as congenital arch malformations or variants of ossification patterns of the cervical spine can be a real pitfall. The unossified cartilages can be mistaken as a fracture. The secondary signs of injury such as soft tissue swelling or presence of asymmetry are helpful to differentiate fracture line from unossified cartilage [9].

Anterior atlas fractures may remain occult in plain radiographs, especially considering the challenge to obtain an open-mouth view in a child with cervical muscle spasm and limited neck movement [2]. Even though the first line of investigation is normal further investigation should be prompted when there is suspicion of fracture. Kapoor et al. described a case when initial conventional radiographs showed no evidence of injury, and a CT scan days later prompted by persistent pain and subsequent representation demonstrated a displaced atlas fracture [9]. Suss in 1983 emphasized the pitfall of the lateral “pseudospread” of C1 in relation to C2 on anteroposterior plain radiographs. Because the atlas initially grows faster than the axis, the normal “spread” of the atlas may be read mistakenly as a disruption of the atlas ring, when it is actually within normal limits [10].

Mechanism of injury, neck pain, head tilt, and decreased range of motion should alert the clinician to the possibility of atlas fracture. CT scan or MRI should be performed even if the plain radiographs showed no abnormality, and higher cervical vertebrae and synchondrosis should be viewed carefully not to miss any pathology. Reconstructed multiplanar MIP and volume rendered images may aid interpretation and increase diagnostic confidence in this potentially difficult clinical scenario.
